# SARS-CoV-2 Omicron BA.5 Infections in Vaccinated Persons, Rural Uganda

**DOI:** 10.3201/eid2901.220981

**Published:** 2023-01

**Authors:** Joseph Mugisha, Bernard Mpairwe, Robert Newton, Matthew Cotten, My V.T. Phan

**Affiliations:** Medical Research Council/Uganda Virus Research Institute and London School of Hygiene & Tropical Medicine, Uganda Research Unit, Entebbe, Uganda (J. Mugisha, B. Mpairwe, R. Newton, M. Cotten, M.V.T. Phan);; University of York, York, United Kingdom (R. Newton);; Medical Research Council–University of Glasgow Center for Virus Research, Glasgow, Scotland, United Kingdom (M. Cotten)

**Keywords:** COVID-19, SARS-CoV-2, Omicron, infections, Uganda, respiratory infections, zoonoses, coronavirus disease, severe acute respiratory syndrome coronavirus 2, viruses

## Abstract

We describe a cluster of COVID-19 breakthrough infections after vaccination in Kyamulibwa, Kalungu District, Uganda. All but 1 infection were from SARS-CoV-2 Omicron strain BA.5.2.1. We identified 6 distinct genotypes by genome sequencing. Infections were mild, suggesting vaccination is not protective against infection but may limit disease severity.

The SARS-CoV-2 Omicron variant BA.5 was initially reported in South Africa in late February 2022 (*1*). The BA.5 variant, and especially the subvariant BA.5.2.1, has now spread to at least 104 countries globally; 197,425 genomes had been reported in GISAID (http://www.gisaid.org) as of September 16, 2022. The BA.5 spike protein shares substitutions with earlier Omicron variants but includes some of the Delta variant immune evasion changes. The BA.4/BA.5 viruses are reported to escape earlier Omicron immune responses, and vaccination does not fully block infection but may limit severity of disease (*2**–**5*). Infection of vaccinated persons (breakthrough infections) with SARS-CoV-2 strains is known, and such infections were reported recently among a highly vaccinated community within the US Embassy in Uganda (*6*). The frequency and outcomes of BA.5 vaccine breakthrough infections, both in Uganda and globally, are yet to be determined.

The Medical Research Council Unit in Uganda maintains a rural population cohort in Kyamulibwa, Kalungu District, southwestern Uganda (*7*). Unit staff were vaccinated as soon as vaccines were available in the country (March 2021), and most received at least 2 doses of COVID-19 vaccine by June 2021, with ongoing efforts for booster vaccination rolling out in the country. Staff members who had any symptoms indicating respiratory infections, including COVID-19, were routinely tested using Abbott’s Panbio COVID-19 antigen rapid tests (Abbott, https://www.abbott.com). If cases of COVID-19 were detected, all staff were tested to detect asymptomatic cases. During such routine testing of staff members, a cluster of SARS-CoV-2 infections among vaccinated staff was detected. Test positivity during this period of infection was 18.5% (12 positive from 65 staff members tested), which was in the range of previous infection waves (January 3–10, 2022: 11.7%; June 6–14, 2021: 32.5%; November 30−December 1, 2020: 19.3%). Most infected staff members had mild symptoms, and all cases were quickly resolved (Appendix Table). 

We performed sequencing by methods previously described (*8*). Nine cases yielded full genome SARS-CoV-2 sequences that we lineage-typed using Nextclade (*9*) and Pangolin (*10*) software; 8 of the 9 genomes were from the BA.5 lineage and 1 was BA.2.31, all variants within the Omicron variant-of-concern lineage. Although all 9 genomes belonged to the Omicron lineage, we detected 6 distinct subvariants (Figure). Genomes from cases 1, 2, 7, and 10 (all BA.5.2.1) were identical, suggesting a common infection source for these 4 cases. However, genomes for cases 4, 6, 11, and 12 genomes (also BA.5.2.1) were distinct from cases 1, 2, 7, and 10 and from each other, differing by 2–5 nt changes. The case 5 genome (BA.2.31) represents a 6th virus source for the cluster of breakthrough infections. 

**Figure:**
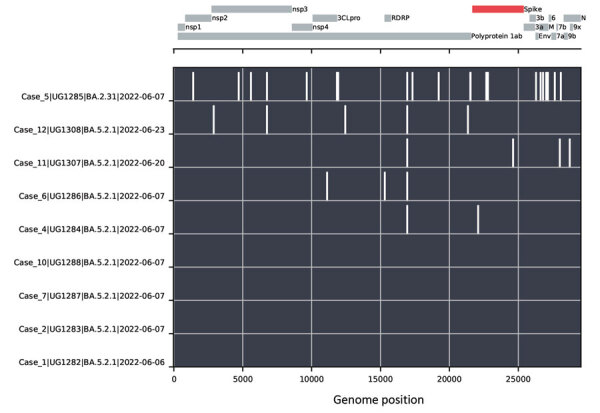
Nucleotide changes between SARS-CoV-2 genomes from a cluster of COVID-19–positive persons in Kyamulibwa, Kalungu District, rural Uganda. The lower portion of the chart shows nucleotide differences from the case 1 genome, plotted as white bars. The absence of bars in the BA.5.2.1 genomes from cases 1, 2, 7, and 10 indicates identical sequences. Case 5 was determined to be lineage BA.2.31, and cases 4, 6, 11, and 12 genomes demonstrated virus variants of lineage BA.5.2.1 distinct from the genomes from cases 1, 2, 7, and 10. A schematic of the SARS-CoV-2 genome is shown in the top portion of the chart, with protein coding regions marked.

The detection of 5 distinct BA.5.2.1 sublineages found in Kalungu District in a short time period indicates multiple BA.5 sublineages were already circulating in other parts of Uganda and demonstrates the speed of movement of SARS-CoV-2. Uganda reported an increase in COVID-19 cases during this period, and both BA.5.2.1 and BA.2.31 virus strains potentially contributed to this increase in infections. Of note, 9 of the 12 COVID-19–positive staff members in this report routinely traveled on shared unit vehicles to and from Masaka or Kampala, which might account for the virus spread. In addition, the unit travel records show shared vehicle usage, suggesting a likely but not confirmed source of infection for cases 2, 7, and 10. The 3 infected staff members whose testing results did not yield sufficient PCR products for sequencing were asymptomatic, suggesting low viral loads (Appendix Table).

Many countries have reported increasing COVID-19 cases with BA.4 or BA.5 and derivatives as a major identified lineage. The global trend toward relaxed travel and quarantine restrictions and the mild infections in vaccinated and previously infected individuals might help enable global movement of these variants. This probably is evidenced by the timing of BA.5 appearance in rural Uganda within weeks of the variant being initially reported in other parts of the world (South Africa in late February 2022, Germany in mid-March 2022, the United States in late March 2022, Portugal in early April 2022, and Uganda in early June 2022).

In conclusion, the detection of 6 distinct sublineages of SARS-CoV-2 (5 of BA.5.2.1 and 1 of BA.2.31) in Kyamulibwa, Kalungu District, Uganda, within a short period indicates substantial diversity of and rapid movement of these viruses into and within Uganda. Combined with recent increases in reported SARS-CoV-2 infections throughout the country, our findings emphasize the need for vigilance, surveillance, and continued testing in this rural community and throughout the country. The mild nature of symptoms in these 12 cases, and in many vaccinated persons, reinforces the importance of community vaccination efforts.

Appendix Additional information on SARS-CoV-2 Omicron BA.5 infections in vaccinated persons, rural Uganda.
