# Supplementary material for: SARS-CoV-2 Omicron BA.5 Infections in Vaccinated Persons, Rural Uganda
Source: Emerg Infect Dis. 2023 Jan;29(1):224–6. doi: 10.3201/eid2901.220981 (PMC9796194; doi:10.3201/eid2901.220981)
Supplement: Appendix — Additional information on SARS-CoV-2 Omicron BA.5 infections in vaccinated persons, rural Uganda. [file 22-0981-Techapp-s1.pdf]

Article DOI: http://doi.org/10.3201/eid2901.220981

SARS-CoV-2 Omicron (BA.5) Infections in Vaccinated Persons, Rural Uganda

Appendix Table. Epidemiologic, demographic, and genomics features from a cluster of COVID-19–positive US Embassy staff members in Kyamulibwa, rural Uganda.

Case no.	Age, y	Gender	Vaccination dose 1	Vaccination dose 2	Vaccine booster	Previous SARS-CoV-2 infections	Travel history (eg, contact with Kampala)	Used Unit bus to Kampala	Shared Unit car to Masaka	Other epi links	Date of disease onset	Symptoms	Outcome	Co-morbidities	Sample collection date	Genome lineage
1	31	F	Mar-21	Jun-21	No	No	Internal travel to Masaka	No	Yes		04-Jun-22	Cough, runny nose, sore throat, chills	Recovered	None	06-Jun-22	BA.5.2.1
2	30	F	Mar-21	Jun-21	No	No	Internal travel to Kampala	Yes	No	Shared office with case 6	06-Jun-22	Runny nose, sore throat, red eyes	Recovered	None	07-Jun-22	BA.5.2.1
3	29	F	Jul-21	Sep-21	No	No	Internal travel to Masaka	No	Yes	Shared office with case 5	Asympt	Asympt	Recovered	None	07-Jun-22	Insufficient material to sequence
4	54	M	Mar-21	Jun-21	No	No	Local travel within Kyamulibwa	No	No		05-Jun-22	Runny nose, fever, chills	Recovered	None	07-Jun-22	BA.5.2.1
5	29	F	Jun-21	Sep-21	No	No	Internal travel to Kampala	Yes	No	Shared office with case 3	06-Jun-22	Cough, runny nose	Recovered	None	07-Jun-22	BA.2.31
6	53	M	Mar-21	Jun-21	No	No	Local travel within Kyamulibwa	No	No	Shared office with case 2	04-Jun-22	Cough, runny nose	Recovered	None	07-Jun-22	BA.5.2.1
7	43	M	Mar-21	Jun-21	No	Yes	Internal travel to Kampala	Yes	No		04-Jun-22	Cough, runny nose, fever, headache	Recovered	Severe allergies	07-Jun-22	BA.5.2.1
8	34	F	Mar-21	Jun-21	No	No	Internal travel to Kampala	Yes	No		Asympt	Asympt	Recovered	None	07-Jun-22	Insufficient material to sequence
9	42	F	Jun-21	No	No	Yes	Local travel within Kyamulibwa	No	No	Cooked and served lunch to station staff.	Asympt	Asympt	Recovered	None	07-Jun-22	Insufficient material to sequence
10	52	M	Mar-21	Jun-21	No	No	Internal travel to Kampala	Yes	No		30-May-22	Cough, runny nose, general weakness, chills	Recovered	None	07-Jun-22	BA.5.2.1
11	29	F	Mar-21	Jun-21	No	Yes	Internal travel to Kampala	Yes	No		19-Jun-22	Flu-like symptoms, malaise	Recovered	None	20-Jun-22	BA.5.2.1
12	42	F	May-21	Jul-21	No	No	Internal travel to Masaka	No	Yes		21-Jun-22	Flu-like symptoms, malaise	Recovered	None	23-Jun-22	BA.5.2.1

Vaccination was with the AstraZeneca COVID-19 vaccine (https://www.astrazeneca.com). Genome lineage determined from near complete genome sequence using Nextclade (*1*) and Pangolin (*2*). Asympt, asymptomatic.

References

1. Aksamentov I, Roemer C, Hodcroft E, Neher R. Nextclade: clade assignment, mutation calling and quality control for viral genomes. [cited 2022 Jun 13]. J Open Source Softw. 2021;6:3773. https://doi.org/10.21105/joss.03773

2. O'Toole Á, Scher E, Underwood A, Jackson B, Hill V, McCrone JT, et al. Assignment of epidemiological lineages in an emerging pandemic using the pangolin tool. Virus Evol. 2021 Jul 30;7(2). doi: 10.1093/ve/veab064 PMID: 34527285
